# Dr. Forbes Royle

**Published:** 1858-05

**Authors:** 


THE news of the death of Dr. FORBES ROYLE will be received with regret in this country, where his work on Materia Medica made his name well known to the profession. He was a graduate in medicine of the University of Munich, a Fellow of the Royal Society of London, and formerly Professor of Materia Medica at King's College. Early in life he practiced in India, with whose material resources, especially those of the vegetable kingdom, he is said to have been very familiar.

